# A Novel *Alu* Element Insertion in *ATM* Induces Exon Skipping in Suspected HBOC Patients

**DOI:** 10.1155/2023/6623515

**Published:** 2023-04-04

**Authors:** Janin Klein, Aldrige B. Allister, Gunnar Schmidt, Annette Otto, Kai Heinecke, Jördis Bax-Knoche, Carmela Beger, Sarah Becker, Stephan Bartels, Tim Ripperger, Jens Bohne, Thilo Dörk, Brigitte Schlegelberger, Winfried Hofmann, Doris Steinemann

**Affiliations:** ^1^Department of Human Genetics, Hannover Medical School, Hannover, Germany; ^2^MVZ Labor Krone GbR, Bad Salzuflen, Bielefeld, Germany; ^3^Department of Pathology, Hannover Medical School, Hannover, Germany; ^4^Department of Virology, Hannover Medical School, Hannover, Germany; ^5^Department of Gynaecology and Obstetrics, Hannover Medical School, Hannover, Germany

## Abstract

The vast majority of patients at risk of hereditary breast and/or ovarian cancer (HBOC) syndrome remain without a molecular diagnosis after routine genetic testing. One type of genomic alteration that is commonly missed by diagnostic pipelines is mobile element insertions (MEIs). Here, we reanalyzed multigene panel data from suspected HBOC patients using the MEI detection tool *Mobster*. A novel *Alu* element insertion in *ATM* intron 54 (ATM:c.8010+30_8010+31insAluYa5) was identified as a potential contributing factor in seven patients. Transcript analysis of patient-derived RNA from three heterozygous carriers revealed exon 54 skipping in 38% of total *ATM* transcripts. To manifest the direct association between the *Alu* element insertion and the aberrant splice pattern, HEK293T and MCF7 cells were transfected with wild-type or *Alu* element-carrying minigene constructs. On average, 77% of plasmid-derived transcripts lacked exon 54 in the presence of the *Alu* element insertion compared to only 4.7% of transcripts expressed by the wild-type minigene. These results strongly suggest ATM:c.8010+30_8010+31insAluYa5 as the main driver of *ATM* exon 54 skipping. Since this exon loss is predicted to cause a frameshift and a premature stop codon, mutant transcripts are unlikely to translate into functional proteins. Based on its estimated frequency of up to 0.05% in control populations, we propose to consider ATM:c.8010+30_8010+31insAluYa5 in suspected HBOC patients and to clarify its role in carcinogenesis through future epidemiological and functional analyses. Generally, the implementation of MEI detection tools in diagnostic sequencing pipelines could increase the diagnostic yield, as MEIs are likely underestimated contributors to genetic diseases.

## 1. Introduction

Genetic testing based on next-generation sequencing (NGS) of individuals with an increased risk of developing breast and/or ovarian cancer (HBOC) is routine practice. The identification of (likely) pathogenic germline variants (PVs) in HBOC-associated genes has clinical implications on the management of carriers and their family members at risk. Breast cancer (BC) surveillance programs, risk-reducing salpingo-oophorectomy, mastectomy, and/or treatment with poly ADP-ribose polymerase inhibitors based on positive outcomes in clinical trials are offered to patients tested positive for PVs in HBOC-associated genes [1].

The German Consortium for HBOC (GC-HBOC) defined 10 HBOC core susceptibility genes that are commonly included in routine genetic testing: *ATM*, *BRCA1*, *BRCA2*, *BRIP1*, *CDH1*, *CHEK2*, *PALB2*, *RAD51C*, *RAD51D*, and *TP53* [2]. Proteins encoded by these genes participate in the homologous recombination repair pathway responsible for error-free DNA double-strand break repair, regulate cell-cycle checkpoints, and contribute to cell-cell adhesions [3–5]. In German HBOC patients subjected to genetic analysis, PVs are identified in *BRCA1* (~10%) and *BRCA2* (~6%), followed by *CHEK2* (~3%), *ATM* (~1%), and *PALB2* (~1%) [6]. The prevalence of PVs for each of the remaining HBOC genes tested is less than 1% [7]. Concluding, in the vast majority of the families meeting the inclusion criteria for genetic testing no PV is identified in any of the HBOC susceptibility genes after routine genetic diagnostics.

The missing heritability in families with a history of HBOC may have different genetic causes: PVs in genes not tested routinely in HBOC families [8], polygenic susceptibility [9], disease-associated copy number variations not characterized by routine sequencing methods [10], variants in regulatory elements [11], inherited epigenetic silencing [12], or spliceogenic variants [13, 14]. In addition, mobile element insertions (MEIs) have gained interest in the field of cancer and genetic predispositions in recent years. While 42% of the human genome consists of mobile elements (MEs) [15], only a small fraction (<0.05%) of them remain active. All active elements are retrotransposons and belong to subfamilies of Long Interspersed Nuclear Elements 1, *Alu,* and Short interspersed element-variable number tandem repeat-Alus (SVA) elements. The insertion of new ME copies may have serious consequences when disrupting protein-coding genes or functional elements [16].

Pathogenic MEIs have been detected in multiple cancer-associated genes, including HBOC susceptibility genes [17, 18]. In a large cohort study [19], enriched for HBOC patients, 45.9% of all pathogenic MEIs found were located in *BRCA2.* Therefore, MEIs detection might be especially important for patients with suspected BRCA2-associated cancer predisposition such as HBOC, familial prostate, or pancreatic adenocarcinoma as well as Fanconi's anemia.

With the rise of NGS, the sensitivity of MEI detection has significantly increased if adequate bioinformatic tools and pipelines are used. This relatively recent progression implicates that MEI detection is still in the early stages and that its importance might be currently underestimated.

One bioinformatic tool to predict MEIs is *Mobster*. *Mobster* can detect novel MEIs in NGS data by analyzing discordant read pairs and clipped reads. Sequences that do not align with the reference genome as expected are compared to consensus sequences of MEs. Among current MEI detection tools, *Mobster* has proven to have a low false discovery rate and a high recall rate for L1 and *Alu* element insertions [20]. Using *Mobster*, we reanalyzed a cohort of more than 300 suspected HBOC patients who did not harbor a (likely) PV after multigene panel sequencing. After filtering and prioritizing the most likely candidates that may predispose or contribute to HBOC, we characterized one predicted *Alu* element insertion in *ATM* intron 54 on DNA and RNA levels in seven unrelated patients. The Alu insertion leads to partial exon 54 skipping, thereby introducing a frameshift and premature termination of translation.

## 2. Results

### 2.1. Characterization and Frequency of ATM:C.8010+30_8010+31insAluYa5

With the aim to identify the genetic cause in PV-negative, suspected HBOC families, we reanalyzed 303 multigene panel datasets with the MEI detection tool *Mobster*. Families fulfilled the inclusion criteria of GC-HBOC (https://www.konsortium-familiaererbrustkrebs.de/betreuungskonzept/molekulare-diagnostik/indikationen-gentest/), underwent genetic testing by multigene panel sequencing at Hannover Medical School (MHH) and were negative for PVs (i.e., classes IV and V single nucleotide variants and copy number variants) in the genes *ATM*, *BAP1*, *BARD1*, *BRCA1*, *BRCA2*, *BRIP1*, *CDH1*, *CHEK2*, *MLH1*, *MSH2*, *MSH6*, *NBN*, *PALB2*, *PMS2*, *PTEN*, *RAD51C*, *RAD51D*, *STK11*, and *TP53* according to GC-HBOC and/or American College of Medical Genetics and Genomics/Association for Molecular Pathology guidelines. Events with a predicted insertion site within 100 bp were pooled, resulting in 75 unique MEI predictions (pMEIs). On average, each patient harbored a mean of 1.2 (0-11) pMEIs, with a median of one pMEI per patient.

Focusing on pMEIs in HBOC core genes (i.e., *ATM*, *BRCA1*, *BRCA2*, *BRIP1*, *CDH1*, *CHEK2*, *PALB2*, *RAD51C*, *RAD51D*, and *TP53*), seven unique pMEIs in 19/303 (6.3%) individuals remained for further analysis: six located in *ATM* and one in *CHEK2*.

**Figure 1:**
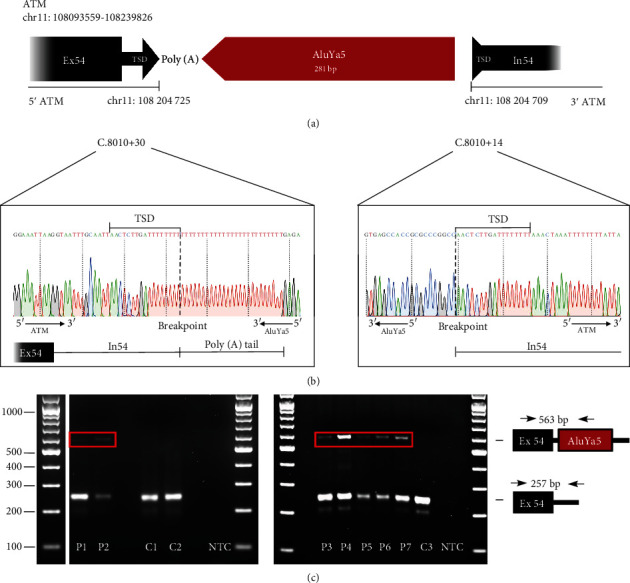
*AluYa5* element insertion confirmed in seven patients (six patients and one additional patient-derived LCL (P7). (a) Schematic representation of *AluYa5* element insertion in *ATM* intron 54 (chr11(GRCh37):g.108204725insAluYa5; NM_0000513:c.8010+30_8010+31insAluYa5). Not drawn to scale. (b) Sanger sequencing electropherogram across breakpoints of the *AluYa5* element insertion after TA cloning showing the insertion breakpoint and the TSD of 17 bp. (c) Gel electrophoresis of PCR products from patients 1-7 (P1-7) and three control individuals (C1-3) using a forward primer located in *ATM* exon 54 and a reverse primer in *ATM* intron 54 showing the expected wild-type band of 257 bp as well as the additional band in the patients with the *AluYa5* element insertion of 563 bp. GRCh37/hg19 was used as reference genome. Bp: basepairs; Ex: exon; In: intron; NTC: no template control; TSD: target site duplication.

One of the seven unique pMEIs, a predicted Alu insertion in *ATM* intron 54, was prioritized for validation, while for the other six pMEIs, read alignments suggested false positive calls, polymorphic events, or sequencing artefacts (Figure 1(a), Supplementary Table 1, Supplementary Figure 1). The selected MEI was predicted in 2/303 (0.7%) nonrelated patients and, in addition, in four patients of an external cohort of 242 suspected HBOC cases. In the latter ones, insertions were indicated based on the routine diagnostic pipeline and confirmed by *Mobster*.

The validation of the insertion in *ATM* intron 54 by PCR and subsequent sequencing revealed a complete, inverted *AluYa5* element sequence at chr11(GRCh37):g.108204725insAluYa5; NM_000051.3:c.8010+30_8010+31insAluYa5. A target site duplication of 17 bp (5′-AACTCTTGA(T)_8_-3′) was observed in all individuals (Figures 1(b) and 1(c)). The core *Alu* sequence of 281 bp was identical in all subjects and aligned to the *AluYa5* element consensus sequence with two mismatches (Supplementary Figure 2). The length of the poly(A)-tail could not be determined with certainty due to polymerase slippage events during amplification and was estimated to be 16-25 bp. In addition, we acquired the lymphoblastoid cell line (LCL) of one patient described in the literature to carry a strikingly similar *Alu* element insertion in *ATM.* PCR and direct sequencing confirmed its match to the *AluYa5* element insertion found in our index patients (patient 7; [21]).

After seven HBOC-risk patients were identified to carry the ATM:c.8010+30_8010+31insAluYa5, we aimed to determine its frequency in a control population as an indicator for its clinical relevance. For this purpose, publicly available whole genome data produced as part of the 1000 Genomes Project [https://www.internationalgenome.org/data-portal/data-collection/30x-grch38]; [22]] were screened for ATM:c.8010+30_8010+31insAluYa5 with *Mobster*. One of 2984 subjects in the public dataset was identified as a carrier resulting in an approximated allele frequency of 0.02%. Interestingly, a second MEI detection tool, *SCRAMble*, identified two additional samples with ATM:c.8010+30_8010+31insAluYa5 in the dataset altering the estimated allele frequency to 0.05%. In contrast, *SCRAMble* only predicted the insertion in four of six index patients.

### 2.2. Semiquantitative Transcript Analysis of Patient RNA Reveals Exon 54 Skipping

**Figure 2:**
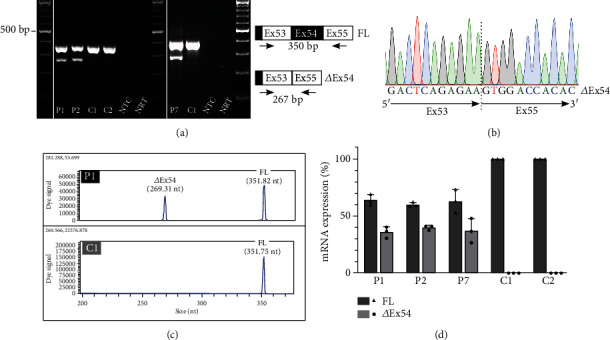
Mutant transcript missing *ATM* exon 54 identified and semi-quantified in patient-derived RNA. (a) Gel electrophoresis RT-PCR products from patients 1, 2, and 7 (P1, P2, and P7) and two control individuals (C1-2) using a forward primer located in *ATM* exon 53 and a reverse primer in *ATM* exon 55. The exon 54-skipped mutant transcript (*Δ*ex54) of 267 bp next to the full-length (FL) transcript of 350 bp is present in *ATM* insAluYa5-carriers and absent in controls. (b) Sanger sequencing electropherogram of the mutant transcripts across the exon 53-exon 55 junction. (c) Capillary electrophoresis of RT-PCR products of patient 1 (P1) and one control sample (C1) depicting the full-length (FL) and mutant (*Δ*ex54) transcripts in the patient, and only FL transcript in the control. (d) Capillary electrophoresis results are shown as bar diagrams depicting the percentage of full-length (FL) and mutant (*Δ*ex54) transcripts in patients 1, 2, and 7 (P1, P2, and P7) and two control individuals (C1-2) next to each other. Nonsense-mediated decay was inhibited by cycloheximide treatment. Data are represented as mean ± SD of three independent experiments. Bp: basepairs; Ex: exon; nt: nucleotides; NTC: no template control; NRT: no reversetranscriptase control.

Lymphocytic RNA was available from patients 1, 2, and 7 to perform transcript analysis. Using reverse transcriptase (RT)-PCR, a mutant transcript missing exon 54 was obtained in *ATM* insertion carriers next to the full-length transcript, but not controls (Figures 2(a) and 2(b)). Since skipping of exon 54, which is 89 bp in length, is predicted to cause a frameshift and premature stop codon (i.e. ATM:p.(Val2671Serfs^∗^17)), this mutant transcript is unlikely to be translated into a functional protein. To determine if the expression levels of the mutant transcript reach clinical relevance, we performed a semiquantitative transcript analysis by labeling one RT-PCR primer with 5′-cyanine 5 (Cy5) and analyzed RT-PCR products by capillary electrophoresis. In 37.7% (range: 26.7-47.9%) of the total transcripts (Figures 2(c) and 2(d)), exon 54 was missing suggesting a considerable part of the mutant allele-derived RNA to be subjected to aberrant splicing.

### 2.3. Minigene Splicing Assay Identifies ATM:C.8010+30_8010+31insAluYa5 as Driver of Exon 54 Skipping

**Figure 3:**
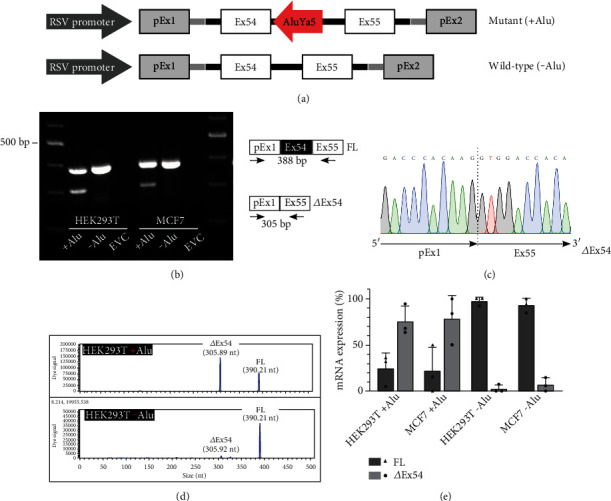
Minigenes with *AluYa5* element insertion predominantly expressed exon 54-lacking mutant transcripts. (a) Schematic representation of the mutant (+ Alu) and wild-type (- Alu) minigenes. The pET01 plasmid-descendent Rous sarcoma virus (RSV) promoter, exons (pEx1-2), and introns are depicted in grey. The *AluYa5* element inserted in *ATM* exon 54 is indicated as red arrow. (b) Gel electrophoresis of RT-PCR products after transfection of HEK293T and MCF7 cells with mutant (+ Alu) and wild-type (- Alu) minigenes show the full-length (FL) transcript of 388 bp and the exon 54-deprived mutant transcript (*Δ*ex54) of 305 bp. (c) Sanger sequencing electropherograms across the junction of the plasmid-descendent exon 1 (pEx1) and *ATM* exon 55 after TA cloning of the mutant transcript. (d) Capillary electrophoresis of RT-PCR products from HEK293T cells transfected with mutant (+ Alu, top) or wild-type (- Alu, bottom) minigenes depicting the full-length (FL) and mutant (*Δ*ex54) transcripts. (e) Capillary electrophoresis results shown as bar diagrams depicting the percentage of full-length (FL) and mutant (*Δ*ex54) transcripts in HEK293T and MCF7 cells after transfection with mutant (+ Alu) or wild-type (- Alu) minigenes. Nonsense-mediated decay was inhibited by cycloheximide treatment. Data are represented as mean ± SD of three independent experiments. Bp: basepairs; EVC: empty vector control; ex: exon; nt: nucleotides.

To verify that *ATM* exon 54 skipping is induced directly by ATM:c.8010+30_8010+31insAluYa5, we performed a minigene splicing assay. We inserted the patient-derived *ATM* exon 54 to exon 55 sequences with and without *AluYa5* element insertion into an ExonTrap pET01 vector, and after transient transfection of HEK293T and MCF7 cell lines, we analyzed ectopic RNA expression (Figure 3(a); Supplementary Figure 3). In line with our previous results, exon 54 skipping was observed in 76.7% (range: 50.1-100%) of total transcripts. The remaining transcripts (23.3% [range: 0-49.9%]) were of full length. The wild-type minigene produced small amounts of mutant transcripts (4.7% [range: 0-15.0%]) and expressed predominantly full-length transcripts (95.3% [range: 85.0-100%]) (Figures 3(b)–3(e)). These results confirm the direct contribution of the *AluYa5* element insertion to induce exon 54 skipping in the majority of expressed RNA in both tested cell lines.

### 2.4. Family History and Cosegregation Analysis

Patients 1-6 fulfilled the GC-HBOC criteria for genetic testing. Patient 1 was first diagnosed with invasive ductal carcinoma of no special type in the left mammary gland at the age of 58 (cT1, cN0) (Supplementary Figure 4A). Several other incidences of BC on the maternal side of the family were documented. In addition, the patient's father was diagnosed with intestinal and pancreatic cancer at the age of 70. No other family members were available for genetic testing. Little information was available for patient 2 and her extended family, who originated from Azerbaijan (Supplementary Figure 4B). Patient 2 was first diagnosed with BC of unknown histology at the age of 38. One of five sisters and one paternal half-aunt also suffered from BC. Besides, one paternal half-uncle suffered from brain cancer. Again, no additional family members were available for genetic testing.

For patient 3, who suffered from BC and was reportedly eligible for HBOC-related testing, the patient and family history were missing. Patient 4 was healthy at the moment of investigation after an ovariectomy at age of 39 years and fulfilled HBOC criteria based on family history. Her mother developed BC (age of onset: 35 years). In addition, the son of patient 4 died from pancreatic adenocarcinoma (age of onset: 53 years). From this sample, genomic DNA (gDNA) isolated from the pancreas carcinoma and healthy colon tissue was available for segregation analysis. Amplification of the region of interest via PCR and visualization via electrophoresis confirmed the presence of ATM:c.8010+30_8010+31insAluYa5 in both tested tissues (Supplementary Figure 5). Sequencing analysis of the son's cancerous tissue revealed an additional heterozygous variant in *ATM* exon 3 (ATM:c.124del:p.His42Ilef^s^∗^^2) which likely causes a loss-of-function of the translated ATM protein (data not shown).

For patient 5 and patient 6, multiple incidences of BC have been reported in the family. Further information on the patients' or families' history was unavailable. Patient 7 has previously been described as a female diagnosed with BC at the age of 56 and was hyperreactive for radiation [21]. No further information about the patient or her family could be obtained.

## 3. Discussion

Identification and characterization of genetic predispositions to HBOC are essential to provide optimal disease surveillance and therapy for patients and their families. Since current diagnostic procedures lead to the detection of PVs in the minority of patients suspected to have HBOC, substantial efforts focus on increasing the diagnostic yield, including the permanent improvement of bioinformatic tools to annotate and classify genomic variants. Here, we reanalyzed multigene panel sequencing data from suspected HBOC patients using the MEI detection tool *Mobster*. One predicted *Alu* insertion in *ATM* intron 54 was confirmed in seven unrelated patients, induced exon skipping of exon 54, and is expected to cause a frameshift variant with a premature stop codon and consequently a truncated, nonfunctional *ATM* protein.

The detection of MEIs remains challenging due to the ME abundance across the human genome and their repeating nature, which causes sequencing and mapping errors [15]. Nonetheless, based on current estimations, MEIs occur *de novo* in 1 of 12-14 live births [23], might be responsible for 0.04% of all genetic diseases [24], and represent up to 0.3% of all disease-causing variants [19]. By implementing MEI detection in routine genetic pipelines, the diagnostic yield might be increased by up to 0.15% [23].

Targeted sequencing, which is currently the standard for genetic testing in most developed countries, produces suboptimal data for MEI detection due to its nonuniformity and limited coverage. While whole genome sequencing will significantly improve the accuracy of MEI detection in the future, bioinformatic tools such as *Mobster* and *SCRAMble* already allow MEI detection from targeted sequencing data but need to be handled with care. Both tools compare clipped sequences with consensus sequences of MEs to identify new insertions but run with slightly different parameters regarding the length of clipped sequences and the number of clipped reads required for an MEI prediction [20, 23]. These differences likely explain the discrepancies in output from *Mobster* and *SCRAMble* regarding ATM:c.8010+30_8010+31insAluYa5. Consequently, we try to consider the application of multiple MEI detection tools for the thorough analysis of sequencing data.

One major obstacle to address before implementing MEI detection into routine diagnostics is the filtering and prioritization of the *in silico* MEI predictions. Contrary to previous reports, the majority of false predictions in our study were not due to false positive calls [25]. Rather, pMEIs were not called in numerous additional samples (false negative) which showed indications of the pMEI in the alignment data at the manual inspection but were not called by *Mobster*. These findings can be explained by either frequent polymorphic events or mapping errors, both of which have little clinical relevance. Consequently, manual validation by read assessment is the key to improve the detection accuracy. In addition, the use of an in-house database could be beneficial to correct the panel- or laboratory-specific artefacts.

The substantial number of false negative pMEIs is likely due to the nonuniformity of coverage of targeted sequencing data, particularly in exon-flanking zones. Interestingly, these zones are enriched for pathogenic MEIs [26]. Multiple examples of disease-associated MEIs within exon-flanking zones have been described previously, most of which induce exon skipping similar to the here described ATM:c.8010+30_8010+31insAluYa5 [27–31]. Since the molecular mechanism of splicing alterations has not been characterized in depth for any of these MEIs, we hypothesize that the insertions, including ATM:c.8010+30_8010+31insAluYa5, disrupt present or introduce novel regulatory splicing elements or alter the RNA secondary structure of the respective region. All of this may modulate the recognition of the nonoptimal nearby 5′ splice site for exon 54 (MaxEnt score of 8.83 compared to 10.86 for the optimal 5′SS). In line with these findings, a small fraction of wild-type transcripts also exhibits exon skipping (Figure 3(e)). In sum, this is in agreement with Lev-Maor et al. [32] who showed alternatively spliced exons are flanked by *Alu* elements more frequently than constitutively spliced exons [32]. In the future, this could be further elucidated by performing minigene depletion assays [33].

The exon 54 skipping induced by ATM:c.8010+30_8010+31insAluYa5 is predicted to cause a frameshift and consequently a premature stop codon after 17 amino acids. While *ATM* exon 54 skipping has not been reported before explicitly, the splice acceptor variant, ATM:c.7928-1G > A (rs1555126163), which was found in an ataxia-telangiectasia patient, but not in controls, is classified as likely pathogenic according to the genetic testing facility Invitae (https://www.invitae.com/en) as reported in ClinVar, since it was assumed to induce exon 54 skipping. Similarly, frameshift and nonsense variants in *ATM* exon 55 [e.g., NM_000051.3:c.8036_8051del p.(Asn2679Serfs^∗^9) (rs587780640) and NM_000051.3:c.8054C > A p.(Ser2685^∗^) (rs2086676230)] have been classified as likely pathogenic, but without functional data as evidence [https://www.ncbi.nlm.nih.gov/clinvar/; [34]]. Therefore, we regard the skipping of exon 54 as deleterious. The pathogenic potential of ATM:c.8010+30_8010+31insAluYa5, however, still needs to be ascertained with care due to its incomplete expressivity established by the minigene splicing assay and penetrance as evidenced by the segregation analysis. The leaky splicing with some 20% of full-length transcript retained might reduce the lifetime risk for cancer in heterozygous carriers compared to classical PVs and, if *in trans* with a classical PV, could result in an atypical form of ataxia-telangiectasia as similarly described in previous publications ([35]; [36]). Patient 4, who is the carrier of ATM:c.8010+30_8010+31insAluYa5, is reportedly healthy. However, the second *ATM* variant in the cancerous tissue of her son is an indication of a substantial loss of functional ATM and the potential involvement of ATM:c.8010+30_8010+31insAluYa5 in tumorigenesis. Pancreatic cancer that occurred among first-degree relatives of two of our seven identified HBOC patients, is known to be associated with pathogenic *ATM* variants [37, 38]. Thus, ATM:c.8010+30_8010+31insAluYa5 might not be the sole driver of HBOC, but the reduced level of functional *ATM* protein may contribute to tumorigenesis [39]. This assumption agrees with the relatively high frequency (0.02-0.05%) of ATM: c.8010+30_8010+31insAluYa5 in control populations which is in the range of the most prevalent reported *BRCA1* and *BRCA2* PVs (0.02-0.03%) [https://gnomad.broadinstitute.org/ (full dataset); [40]]. *ATM* PVs in general (0.4-2.0% in the general population) are relatively frequent which can only be tolerated due to their recessive nature and incomplete penetrance [38, 41]. Although ATM PVs can be generally associated with an increased risk to develop BOC, its degree highly depends on the variant type and location and is difficult to determine [42], particularly for leaky splicing variants. In summary, our study identified and characterized ATM:c.8010+30_8010+31insAluYa5 as a likely contributor to HBOC in seven families. We demonstrated a clinical utility of reanalyzing diagnostic sequencing data for MEI and strongly recommend the implementation of MEI detection tools in routine sequencing bioinformatic pipelines to increase the diagnostic yield in familial cancer predisposition as well as ataxia telangiectasia and gain more insights into MEIs, their prevalence, and pathogenicity.

## 4. Methods

### 4.1. Subjects

All individuals included gave their informed written consent for participating in our study. The study was approved by the local ethics committee (Hannover Medical School, Ethic votes: Nr. 4121 and extension Nr. 8657_BO_K_2019).

Multigene panel sequencing data from 303 nonrelated female BOC patients (including patient 1 and patient 2) who underwent genetic counseling at the Hannover Medical School between 2001 and 2020 were analyzed with the MEI detection tool *Mobster* after regarded negative for PVs (single nucleotide variants and copy number variants) in the genes *ATM*, *BAP1*, *BARD1*, *BRCA1*, *BRCA2*, *BRIP1*, *CDH1*, *CHEK2*, *MLH1*, *MSH2*, *MSH6*, *NBN*, *PALB2*, *PMS2*, *PTEN*, *RAD51C*, *RAD51D*, *STK11*, and *TP53* according to GC-HBOC and/or American College of Medical Genetics and Genomics/Association for Molecular Pathology guidelines. The cohort was first described by Schubert et al. [8] and continuously extended [8].

On average, patients fulfilled two GC-HBOC inclusion criteria and all patients fulfilled at least one (https://www.konsortium-familiaerer-brustkrebs.de/betreuungskonzept/molekulare-diagnostik/indikationen-gentest/). The cohort included 281 patients diagnosed with BC, 17 with OC, and five with BC and OC. The median age at first diagnosis was 43 years ranging from 17 to 74 years.

The analysis was extended by 242 multigene panel datasets from MVZ Labor Krone GbR (Bad Salzuflen/Bielefeld, Germany), ATM:c.8010+30_8010+31insAluYa5 was detected by the routine diagnostic software from Sophia Genetics and confirmed by the MEI detection tool *Mobster* in four patients (patients 3, 4, 5, and 6). Again, all patients fulfilled at least one GC-HBOC inclusion criterium (https://www.konsortium-familiaerer-brustkrebs.de/betreuungskonzept/molekulare-diagnostik/indikationen-gentest/). Unfortunately, more detailed information on patients 3, 5, and 6 and/or their family history could not be obtained due to data protection laws. Patient 4 was reportedly healthy at the moment of investigation but was eligible for the study based on her family history.

Patient 7 has been previously described [21]. We kindly received a sample of the patient's frozen LCL from Professor Detlev Schindler, University of Würzburg. To our knowledge, NGS analysis has not been performed.

For NGS and variant confirmation by Sanger sequencing, germline DNA was isolated from peripheral blood leukocytes according to standard procedures.

### 4.2. *In Silico* Prediction of MEIs

Reference genome GRCh37/hg19 was used throughout the study if not stated otherwise. NGS data was analyzed with the MEI detection tools *Mobster* (version 0.2.4.1) and *SCRAMble* (version 1.0.2) using default settings [20, 23]. All pMEIs within the ten HBOC core genes (i.e., *ATM*, *BRCA1*, *BRCA2*, *BRIP1*, *CDH1*, *CHEK2*, *PALB2*, *RAD51C*, *RAD51D*, and *TP53*) were manually evaluated using Integrative Genomics Viewer v2.12.2 (https://software.broadinstitute.org/software/igv/) by two independent reviewers. To estimate the population frequency, 2984 publicly available whole genome datasets originating from 26 populations aligned to GRCh38/hg38 from the 1000 Genomes Project [https://www.internationalgenome.org/data-portal/data-collection/30x-grch38; [22]] were screened by *Mobster* and *SCRAMble* for ATM:c.8010+30_8010+31insAluYa5. Further information on dataset demographics is not publicly available.

### 4.3. Confirmation of ATM:C.8010+30_8010+31insAluYa5 Insertion

For all study subjects, DNA from peripheral blood leukocytes or LCL was amplified using primers #6418 and #6419 flanking the predicted insert point (Supplementary Table 2). PCR was performed using HotStarTaq DNA Polymerase with QIAGEN PCR Buffer, and Q-Solution (Qiagen, Germany). The thermocycling conditions were chosen as follows: initial denaturation at 95°C for 15 min, 30 cycles of 95°C for 45 sec, 58°C for 45 sec, 72°C for 60 sec, and a final extension at 72°C for 10 min. The PCR products were electrophoresed and bands of interest were excised and extracted using NucleoSpin™ Gel and PCR Clean-up (XS) Kit (Macherey-Nagel, Germany). The isolated and purified PCR products were then introduced into pCR™ 2.1-TOPO™ TA vector using the TOPO™ TA Cloning® Kit (Invitrogen, Germany) following the manufacturer's instructions. After vector preparation with QIAprep Spin Miniprep Kit (Qiagen, Germany), inserted PCR products were Sanger sequenced by Microsynth Seqlab (Microsynth, Germany) using M13-primers. Electropherograms were analyzed using SnapGene® Viewer 5.2.3.

### 4.4. Minigene Splicing Assay

Minigene splicing constructs encompassing the genomic region chr11:108,204,538-108,205,876, which includes exon 54 and 55 of *ATM*, were prepared by PCR amplification from patient DNA using primer #6603 and #6685 carrying *Bam*HI and *Xba*I restriction sites, respectively (Supplementary Table 2). Amplified regions were subcloned into Exontrap vector pET01 (MoBiTec, Germany) in direct orientation. All constructs were verified via Sanger sequencing and endonuclease digestion. HEK293T and MCF7 cells were transfected via cationic lipid-mediated transfection with wild-type and mutant constructs in triplicate. Briefly, 5^∗^10 5 and 7^∗^10 5 HEK293T and MCF7 cells, respectively, were seeded in 12-well plates and directly or reverse transfected using 50-times diluted Lipofectamine 2000 (Invitrogen, Germany) and 1250 ng DNA in equal volumes totaling up to 125 *μ*l per well. Cells were harvested 30 h after transfection. Prior to cell harvest, cells were treated with 50 *μ*g/ml cycloheximide (Sigma-Aldrich, Germany) for 6 hours to inhibit nonsense-mediated decay.

### 4.5. Cell Culture

All cells were cultured at 37°C in a humidified atmosphere with 5% CO_2_, and the medium was replaced every 2-3 days. Cells were split when 70-90% confluence was reached. LCLs were generated from 3-6 ml of fresh whole blood supplemented with EDTA. Briefly, mononuclear cells were isolated and supplemented with Epstein-Barr virus-containing supernatant from B95-8 cells in growth medium: RPMI 1640 supplemented with 10% (*v*/*v*) fetal bovine serum, 1% (*v*/*v*) penicillin/streptomycin, 1% (*v*/*v*) L-glutamine, and 1% 1 M 4-(2-hydroxyethyl)-1-piperazineethanesulfonic acid. After 2 hours of incubation at 37°C, 500 ng/ml cyclosporin A (Sandimmun®; Novartis, Germany) was added. HEK293T cells were cultured in DMEM with 4.5 g/l glucose, 10% (*v*/*v*) fetal bovine serum, 1% (*v*/*v*) penicillin/streptomycin, and 1% (*v*/*v*) sodium pyruvate. MCF7 cells were cultured in DMEM with 4.5 g/l glucose, 10% (*v*/*v*) fetal bovine serum, 1% (*v*/*v*) penicillin/streptomycin, and 10 *μ*g/ml human insulin.

### 4.6. RNA Isolation, cDNA Synthesis, and Transcript Analysis

Total RNA was isolated from cultured cells and frozen cell pellets using Direct-zol RNA Miniprep Kit (Zymo Research, Germany) or NucleoSpin® RNA Mini Kit (Macherey-Nagel, Germany) following the manufacturer's instructions. RNA was reverse transcribed into cDNA using High-Capacity cDNA Reverse Transcription Kit (Thermo Fisher Scientific, Germany) following the manufacturer's instructions with one exception. For RNA from transfected cells, reverse transcription was performed with the plasmid-specific primer #6386 (Supplementary Table 2). For all other reactions, random hexamer primers included in the High-Capacity cDNA Reverse Transcription Kit (Thermo Fisher Scientific, Germany) were used.

Transcript analysis was performed in triplicates by RT-PCR using 5′-Cy5-labeled primer #6605 and primer #6606 for patient-derived RNA or 5′-Cy5-labeled primer #6606 and primer #6387 for minigene-derived RNA (Supplementary Table 2) and subsequent capillary electrophoresis using the Beckman Coulter CEQ 8000 GeXP Genetic Analysis system (Beckman Coulter, Germany) combined with the GenomeLab™ GeXP software v11.0 (SCIEX, USA). The samples were run with D1-labeled Size Standard 600 (Beckman Coulter, Germany). Only peak areas more than three times above the baseline noise were considered. For relative quantification, the total peak area was regarded as the total target mRNA expression and compared to individual peak areas.

### 4.7. NGS Panel Sequencing on FFPE-Based DNA

From a tumor- and a normal tissue bearing paraffin block of patient 4, six 5 *μ*m-thick sections were cut, and tumor infiltrates were enriched by microdissection. DNA was extracted with a Maxwell RSC DNA FFPE Kit (Promega, USA) on a Maxwell RSC instrument according to the manufacturer's recommendations. Subsequently, DNA samples were quantified with a Qubit 2.0 Fluorometer (Invitrogen, Germany) and the Qubit dsDNA HS Assay Kit (Life Technologies, USA). In total, 20 ng of FFPE-DNA was used for NGS sequencing with the Oncomine™ comprehensive v3 assays, covering 161 cancer-related genes including the full-length coding sequence of the *ATM* gene, on an Ion S5 prime sequencer (Thermo Fisher Scientific, USA). Data evaluation and variant annotation were performed with ANNOVAR software and database tools (http://www.openbioinformatics.org/annovar/ and Wang et al. [43]).

## Data Availability

Data are available within the manuscript.

## Supplementary Materials

Supplementary Materials Supplementary 1: Supplementary Table 1: predicted mobile element insertions in HBOC core genes excluded after manual evaluation of read alignments. Supplementary 2: Supplementary Table 2: primers. All primers were purchased from metabion (Germany). Supplementary 3: Supplementary Figure 1: read alignment shows discordant and split reads across the AluYa5 element insertion site in ATM (NM_000051.3) intron 54. Supplementary 4: Supplementary Figure 2: sequences of patients' Alu element inserted in ATM intron 54 aligns to AluYa5 element consensus sequence with two mismatches. Supplementary 5: Supplementary Figure 3: workflow of minigene splicing assay. Supplementary 6: Supplementary Figure 4: family pedigrees of patient 1 and patient 2. Supplementary 7: Supplementary Figure 5: AluYa5 element insertion confirmed via PCR in patient 4.
