# Supplementary material for: A Novel Alu Element Insertion in ATM Induces Exon Skipping in Suspected HBOC Patients
Source: Hum Mutat. 2023 Apr 4;2023:6623515. doi: 10.1155/2023/6623515 (PMC11919196; doi:10.1155/2023/6623515)
Supplement: Supplementary Materials — Supplementary 1: Supplementary Table 1: predicted mobile element insertions in HBOC core genes excluded after manual evaluation of read alignments. Supplementary 2: Supplementary Table 2: primers. All primers were purchased from metabion (Germany). Supplementary 3: Supplementary Figure 1: read alignment shows discordant and split reads across the AluYa5 element insertion site in ATM (NM_000051.3) intron 54. Supplementary 4: Supplementary Figure 2: sequences of patients' Alu element inserted in ATM intron 54 aligns to AluYa5 element consensus sequence with two mismatches. Supplementary 5: Supplementary Figure 3: workflow of minigene splicing assay. Supplementary 6: Supplementary Figure 4: family pedigrees of patient 1 and patient 2. Supplementary 7: Supplementary Figure 5: AluYa5 element insertion confirmed via PCR in patient 4. [file 6623515.f1.zip › sup_figures+tables_legend_final.docx]

**Supplementary Figure 1. Read alignment shows discordant and split reads across the AluYa5 element insertion site in ATM (NM_000051.3) intron 54.**

Screenshot of read alignment after TruSight Cancer panel sequencing of patient 2 (P2) visualized with the Integrative Genomics Viewer v2.12.2 (https://software.broadinstitute.org/software/igv/). Reads were grouped by reference concordance and sorted by start location. Reads within the red box were labeled as discordant reads for which the pair read did not map to the reference as expected. Split reads clearly show the AluYa5 element’s poly(A) tail indicated as red, mismatched bases
(= Ts due to the antisense orientation of the AluYa5 element insertion).

**Supplementary Figure 2. Sequences of patients’ Alu element inserted in ATM intron 54 aligns to AluYa5 element consensus sequence with two mismatches.**

Sequences were aligned using EMBL-EBI tool Clustal Omega [https://www.ebi.ac.uk/Tools/msa/clustalo/; (Sievers et al., 2011)] and visualized by SnapGene Viewer Version 5.2.3. AluYa5 element consensus sequence (DF0000053.4) was obtained from the Dfam database [https://www.dfam.org; (Hubley et al., 2016)].

**Supplementary Figure 3. Workflow of minigene splicing assay.**

The mutant (mut)- and wild-type (wt)-allele were amplified from patient DNA and ligated into the multiple cloning site (MCS) of the pET01 ExonTrap vector flanked by the plasmid-descendent exons pEx1 and pEx2. HEK293T and MCF7 cells were transfected with vectors containing the mutant or wild-type minigene, or empty vector as control. After 24 h, mRNA decay was inhibited with 50 µg/ml cycloheximide for 6 h. Isolated RNA was synthesized into cDNA and analyzed via RT-PCR and electrophoresis. The illustration was created with BioRender (https://biorender.com/).

**Supplementary Figure 4. Family pedigrees of patient 1 and patient 2.**

**A**. Family pedigree of patient 1. **B**. Family pedigree of patient 2. Circles: Female; Squares: Male; Filled solid: Breast cancer; Partially filled: Other type of cancer; Unfilled: Unaffected; Crossed: Deceased. BC: Breast cancer; BIC: Breast and intestinal cancer; BrC: Brain cancer; IPC: Intestinal and pancreatic cancer. The numbers next to the cancer description represent the age of diagnosis. The numbers in brackets represent the age of death. The numbers on the upper left corner of each symbol function as signifiers. An arrow marks the index patients.

**Supplementary Figure 5. AluYa5 element insertion confirmed via PCR in patient 4**

Validation of AluYa5 element insertion in ATM intron 54 (chr11(GRCh37):g.108204725insAluYa5; ATM NM_0000513 c.8010+30_8010+31insAluYa5) via PCR. Gel electrophoresis of PCR products from gDNA from pancreas cancer sample (k) (son of patient 4) and control (gDNA from patient 4 (m)) using a forward primer located in ATM exon 54 and a reverse primer in ATM intron 54 showing the expected wild-type band of 240 bp as well as the additional band in the patients with the AluYa5 element insertion of 560 bp. K: gDNA from pancreas cancer sample (son of patient 4), NTC: negative control, using water as template for PCR, m: gDNA from patient 4 (female).

**Supplementary Table 1.** Predicted mobile element insertions in HBOC core genes excluded after manual evaluation of read alignments.

**Supplementary Table 2.** Primers. All primers were purchased from metabion (Germany).
