# Supplementary material for: A Novel Alu Element Insertion in ATM Induces Exon Skipping in Suspected HBOC Patients
Source: Hum Mutat. 2023 Apr 4;2023:6623515. doi: 10.1155/2023/6623515 (PMC11919196; doi:10.1155/2023/6623515)
Supplement: Supplementary Materials — Supplementary 1: Supplementary Table 1: predicted mobile element insertions in HBOC core genes excluded after manual evaluation of read alignments. Supplementary 2: Supplementary Table 2: primers. All primers were purchased from metabion (Germany). Supplementary 3: Supplementary Figure 1: read alignment shows discordant and split reads across the AluYa5 element insertion site in ATM (NM_000051.3) intron 54. Supplementary 4: Supplementary Figure 2: sequences of patients' Alu element inserted in ATM intron 54 aligns to AluYa5 element consensus sequence with two mismatches. Supplementary 5: Supplementary Figure 3: workflow of minigene splicing assay. Supplementary 6: Supplementary Figure 4: family pedigrees of patient 1 and patient 2. Supplementary 7: Supplementary Figure 5: AluYa5 element insertion confirmed via PCR in patient 4. [file 6623515.f1.zip › Supplementary Table 1.docx]

**Supplementary Table 1.**

| Gene | Location | Predicted insertion point (hg19) | Predicted ME type | No. of samples | Reason for exclusion |
| --- | --- | --- | --- | --- | --- |
| *ATM* | Intron 5 | chr11:108114599 | L1 | 1 | Found in >10 additional samples. Either polymorphic or mapping artefact. |
| *ATM* | Intron 9 | chr11:108121403 | L1 | 2 | Found in >10 additional samples. Either polymorphic or mapping artefact. |
| *ATM* | Intron 19 | chr11:108141955 | L1 | 1 | Found in >10 additional samples. Either polymorphic or mapping artefact. |
| *ATM* | Intron 45 | chr11:108195976 | L1 | 5 | Found in >10 additional samples. Either polymorphic or mapping artefact. |
| *ATM* | Intron 51 | chr11:108202485 | ALU | 1 | False positive. No sign of insertion. |
| *CHEK2* | Intron 1 | chr22:29130813 | L1 | 8 | Found in >10 additional samples. Either polymorphic or mapping artefact. |
