# Supplementary figures and images for: A Novel Alu Element Insertion in ATM Induces Exon Skipping in Suspected HBOC Patients
Source: Hum Mutat. 2023 Apr 4;2023:6623515. doi: 10.1155/2023/6623515 (PMC11919196; doi:10.1155/2023/6623515)

Figure S1

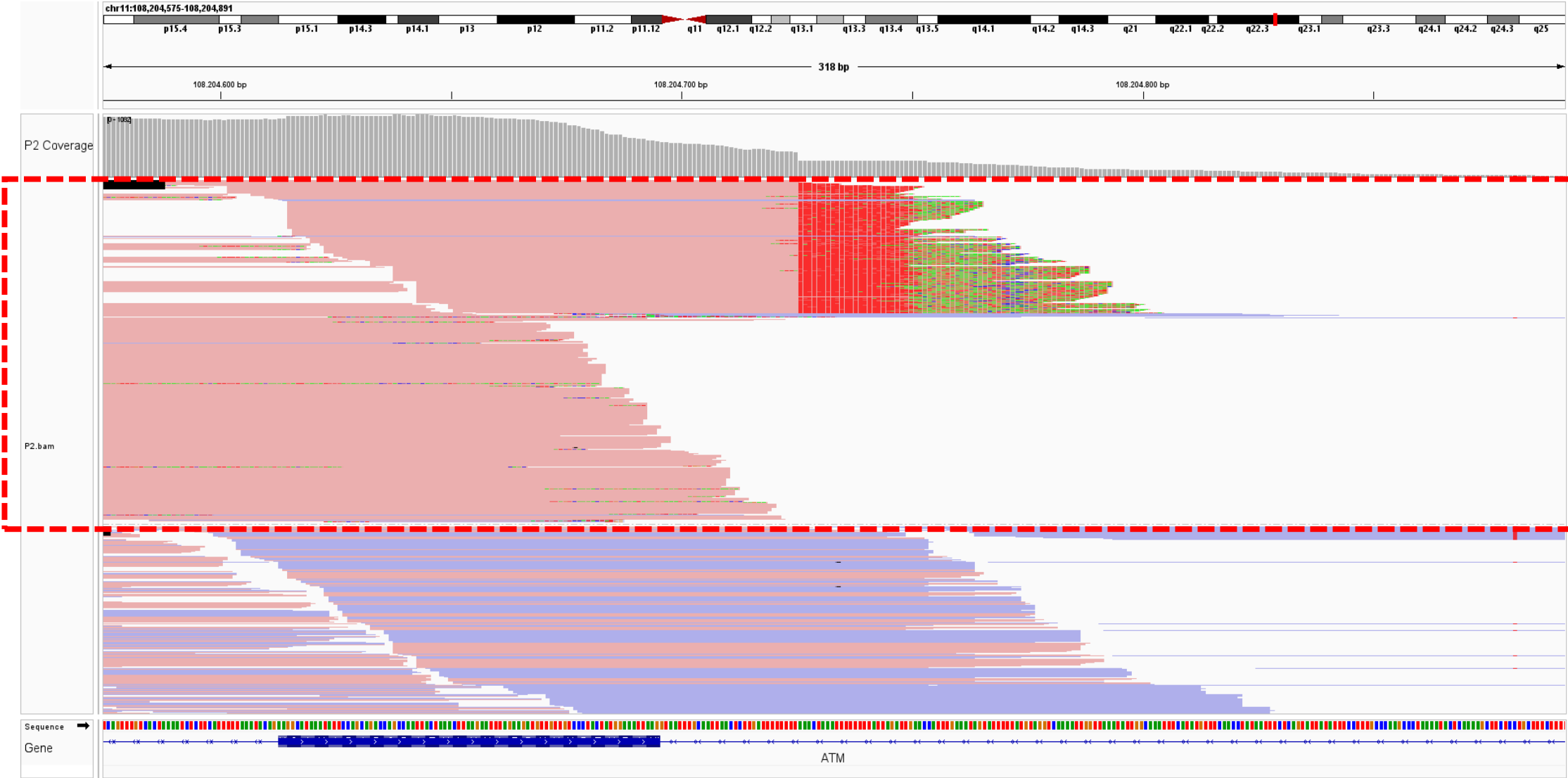

Supplement: Supplementary Materials — Supplementary 1: Supplementary Table 1: predicted mobile element insertions in HBOC core genes excluded after manual evaluation of read alignments. Supplementary 2: Supplementary Table 2: primers. All primers were purchased from metabion (Germany). Supplementary 3: Supplementary Figure 1: read alignment shows discordant and split reads across the AluYa5 element insertion site in ATM (NM_000051.3) intron 54. Supplementary 4: Supplementary Figure 2: sequences of patients' Alu element inserted in ATM intron 54 aligns to AluYa5 element consensus sequence with two mismatches. Supplementary 5: Supplementary Figure 3: workflow of minigene splicing assay. Supplementary 6: Supplementary Figure 4: family pedigrees of patient 1 and patient 2. Supplementary 7: Supplementary Figure 5: AluYa5 element insertion confirmed via PCR in patient 4. [file 6623515.f1.zip › figure s1.pdf]

Figure S2

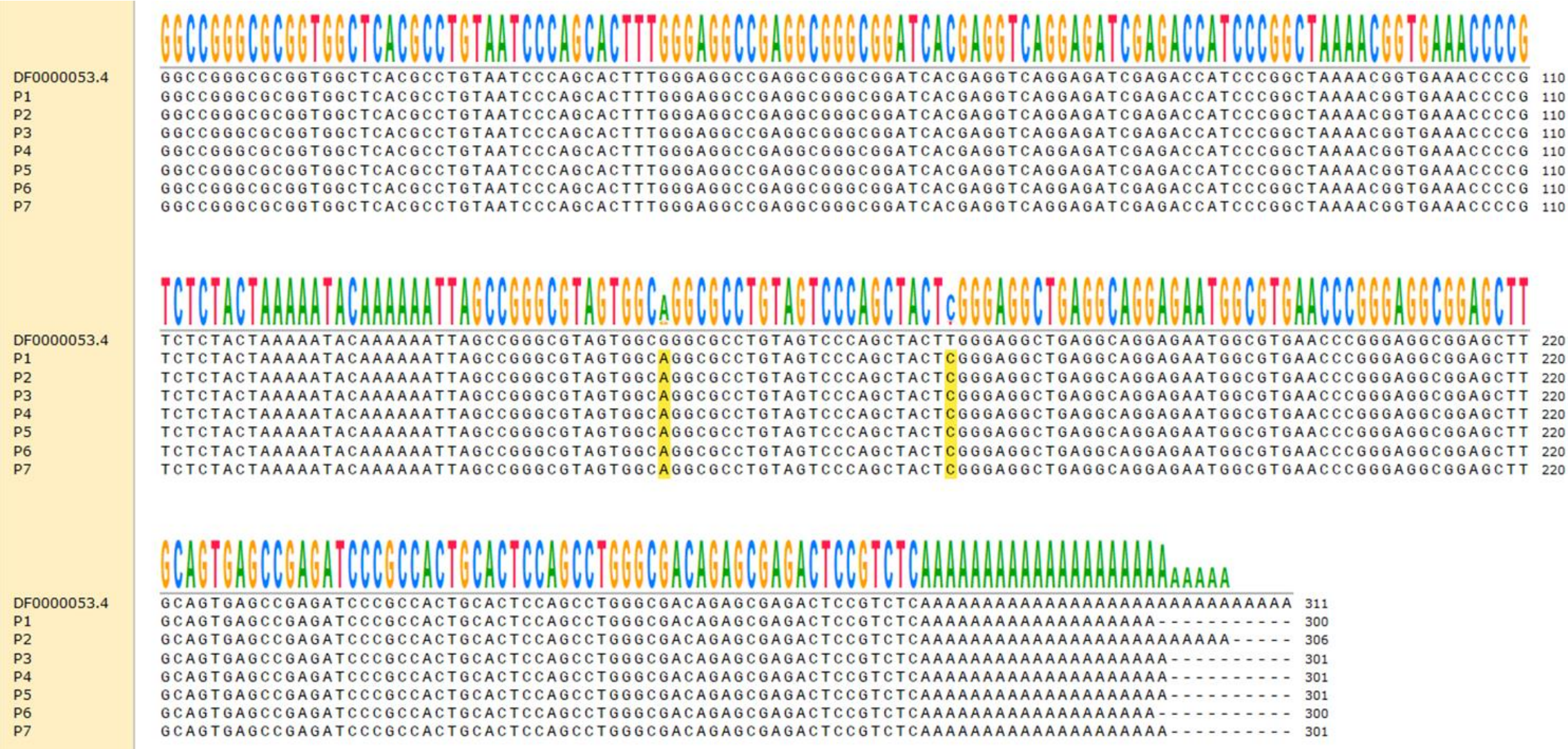

Supplement: Supplementary Materials — Supplementary 1: Supplementary Table 1: predicted mobile element insertions in HBOC core genes excluded after manual evaluation of read alignments. Supplementary 2: Supplementary Table 2: primers. All primers were purchased from metabion (Germany). Supplementary 3: Supplementary Figure 1: read alignment shows discordant and split reads across the AluYa5 element insertion site in ATM (NM_000051.3) intron 54. Supplementary 4: Supplementary Figure 2: sequences of patients' Alu element inserted in ATM intron 54 aligns to AluYa5 element consensus sequence with two mismatches. Supplementary 5: Supplementary Figure 3: workflow of minigene splicing assay. Supplementary 6: Supplementary Figure 4: family pedigrees of patient 1 and patient 2. Supplementary 7: Supplementary Figure 5: AluYa5 element insertion confirmed via PCR in patient 4. [file 6623515.f1.zip › figure s2.pdf]

Figure S4

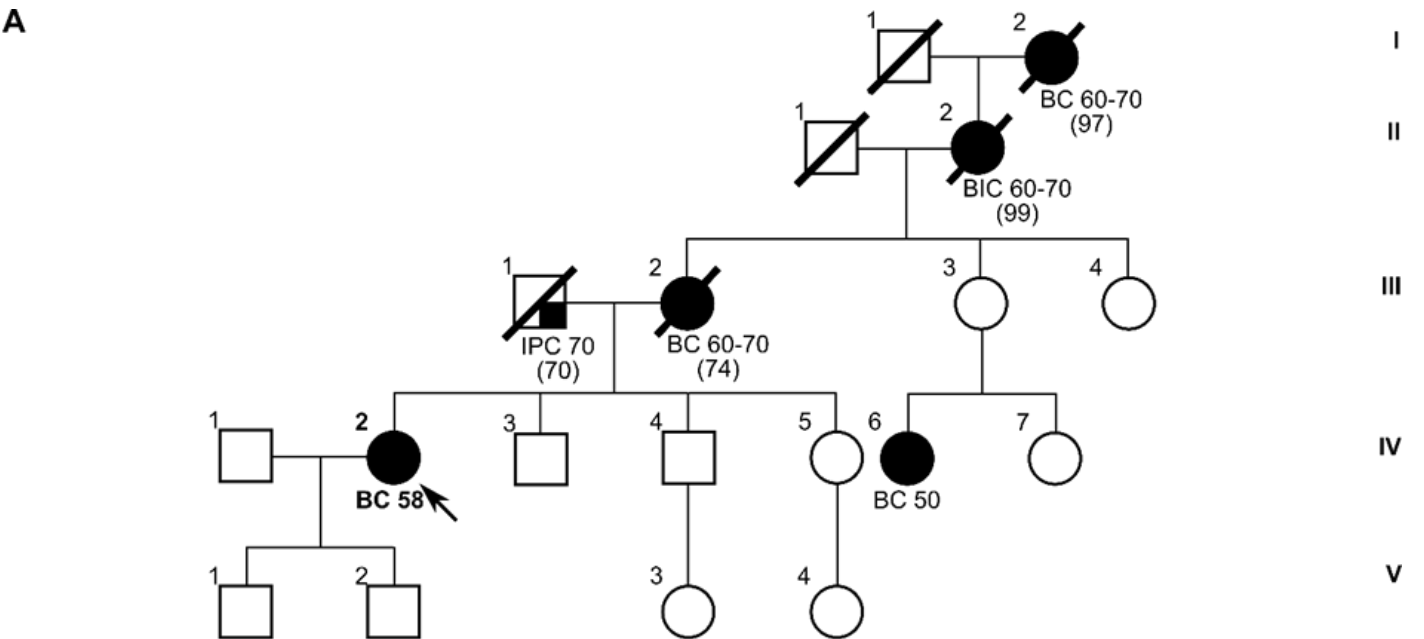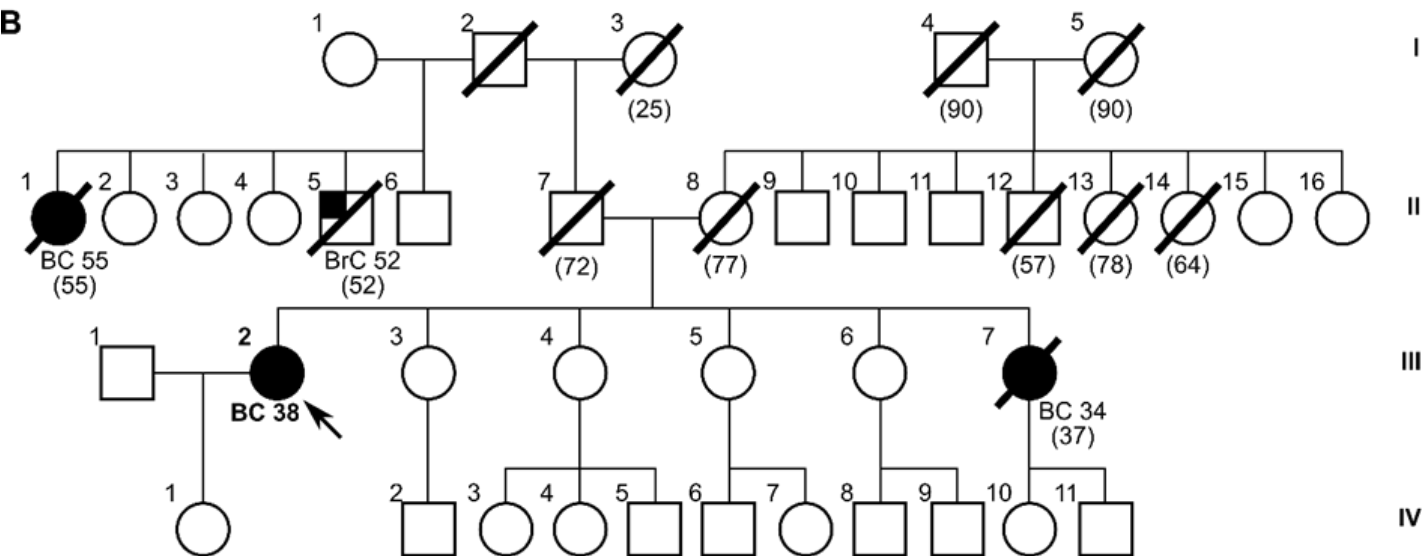

Supplement: Supplementary Materials — Supplementary 1: Supplementary Table 1: predicted mobile element insertions in HBOC core genes excluded after manual evaluation of read alignments. Supplementary 2: Supplementary Table 2: primers. All primers were purchased from metabion (Germany). Supplementary 3: Supplementary Figure 1: read alignment shows discordant and split reads across the AluYa5 element insertion site in ATM (NM_000051.3) intron 54. Supplementary 4: Supplementary Figure 2: sequences of patients' Alu element inserted in ATM intron 54 aligns to AluYa5 element consensus sequence with two mismatches. Supplementary 5: Supplementary Figure 3: workflow of minigene splicing assay. Supplementary 6: Supplementary Figure 4: family pedigrees of patient 1 and patient 2. Supplementary 7: Supplementary Figure 5: AluYa5 element insertion confirmed via PCR in patient 4. [file 6623515.f1.zip › figure s4.pdf]

Figure s5

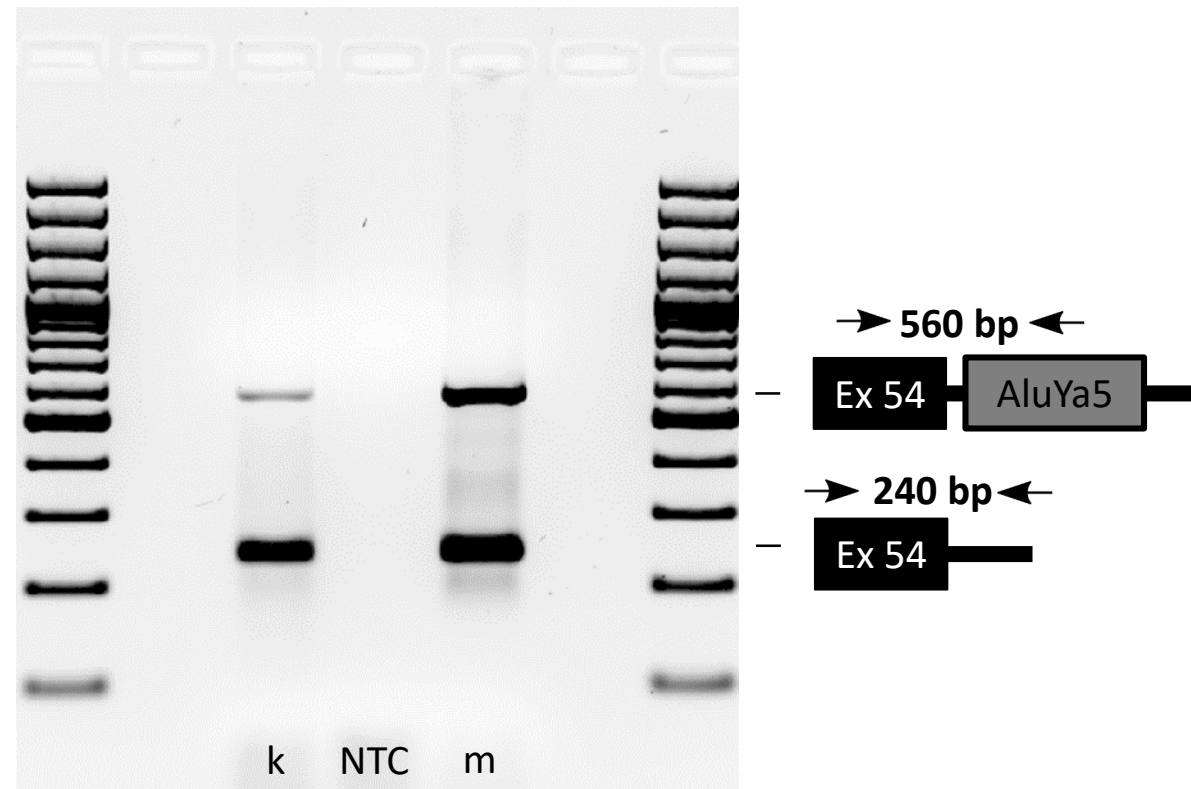

Supplement: Supplementary Materials — Supplementary 1: Supplementary Table 1: predicted mobile element insertions in HBOC core genes excluded after manual evaluation of read alignments. Supplementary 2: Supplementary Table 2: primers. All primers were purchased from metabion (Germany). Supplementary 3: Supplementary Figure 1: read alignment shows discordant and split reads across the AluYa5 element insertion site in ATM (NM_000051.3) intron 54. Supplementary 4: Supplementary Figure 2: sequences of patients' Alu element inserted in ATM intron 54 aligns to AluYa5 element consensus sequence with two mismatches. Supplementary 5: Supplementary Figure 3: workflow of minigene splicing assay. Supplementary 6: Supplementary Figure 4: family pedigrees of patient 1 and patient 2. Supplementary 7: Supplementary Figure 5: AluYa5 element insertion confirmed via PCR in patient 4. [file 6623515.f1.zip › figure s5.pdf]

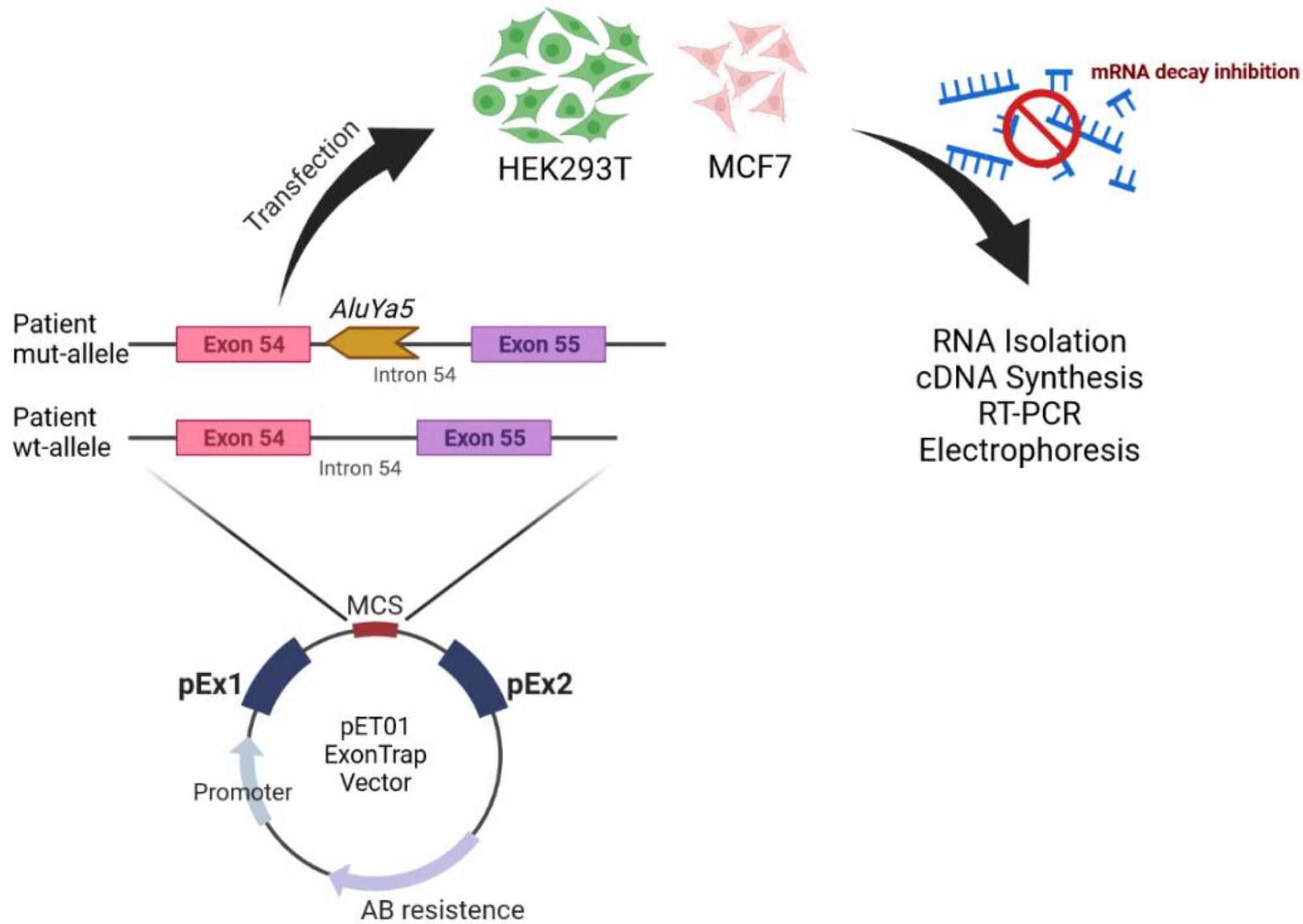

Supplement: Supplementary Materials — Supplementary 1: Supplementary Table 1: predicted mobile element insertions in HBOC core genes excluded after manual evaluation of read alignments. Supplementary 2: Supplementary Table 2: primers. All primers were purchased from metabion (Germany). Supplementary 3: Supplementary Figure 1: read alignment shows discordant and split reads across the AluYa5 element insertion site in ATM (NM_000051.3) intron 54. Supplementary 4: Supplementary Figure 2: sequences of patients' Alu element inserted in ATM intron 54 aligns to AluYa5 element consensus sequence with two mismatches. Supplementary 5: Supplementary Figure 3: workflow of minigene splicing assay. Supplementary 6: Supplementary Figure 4: family pedigrees of patient 1 and patient 2. Supplementary 7: Supplementary Figure 5: AluYa5 element insertion confirmed via PCR in patient 4. [file 6623515.f1.zip › figure_s3.pdf]
